# Land Ecological Security Evaluation of Guangzhou, China

**DOI:** 10.3390/ijerph111010537

**Published:** 2014-10-15

**Authors:** Linyu Xu, Hao Yin, Zhaoxue Li, Shun Li

**Affiliations:** 1 State Key Joint Laboratory of Environmental Simulation and Pollution Control, School of Environment, Beijing Normal University, No. 19 Xinjiekouwai Street, Haidian District, Beijing 100875, China; E-Mails: yinhao@mail.bnu.edu.cn (H.Y.); lizhaoxuesnow@gmail.com (Z.L.); 2 Environmental Information Center, Ministry of Environmental Protection, No.1 Yuhuinanlu, Chaoyang District, Beijing 100029, China; E-Mail: lishun@mep.gov.cn

**Keywords:** Guangzhou, land ecological security, LES supply and demand, material security, spiritual security

## Abstract

As the land ecosystem provides the necessary basic material resources for human development, land ecological security (LES) plays an increasingly important role in sustainable development. Given the degradation of land ecological security under rapid urbanization and the urgent LES requirements of urban populations, a comprehensive evaluation method, named Double Land Ecological Security (DLES), has been introduced with the city of Guangzhou, China, as a case study, which evaluates the LES in regional and unit scales for reasonable and specific urban planning. In the evaluation process with this method, we have combined the material security with the spiritual security that is inevitably associated with LES. Some new coefficients of land-security supply/demand distribution and technology contribution for LES evaluation have also been introduced for different spatial scales, including the regional and the unit scales. The results for Guangzhou indicated that, temporally, the LES supply indices were 0.77, 0.84 and 0.77 in 2000, 2006 and 2009 respectively, while LES demand indices for the city increased in 2000, 2006 and 2009 from 0.57 to 0.95, which made the LES level decreased slowly in this period. Spatially, at the regional scale, the urban land ecological security (ULES) level decreased from 0.2 (marginal security) to −0.18 (marginal insecurity) as a whole; in unit scale, areas in the north and in parts of the east were relatively secure and the security area was shrinking with time, but the central and southern areas turned to be marginal insecurity, especially in 2006 and 2009. This study proposes that DLES evaluation should be conducted for targeted and efficient urban planning and management, which can reflect the LES level of study area in general and in detail.

## 1. Introduction 

There is ample evidence that land ecosystems in many regions have become highly stressed and dysfunctional due to continuous, excessive exploitation and utilization of land resources [1,2,3], which causes billions of dollars in losses per year [4]. With the rapid pace of urbanization and the development of human societies, people living in some urban areas are confronted with land pollution, soil erosion, land desertification, the degradation of forest vegetation and the reduction of biodiversity, which increasingly threatens urban land ecological security (LES). Escalating concerns over urban land ecological security are prompting the need for guidelines for the redesign of cities and development models in ways that seek to help to secure the resources necessary for ecological and material guarantees. Planners and policy makers are in growing need of significant new data and scientific knowledge about the state of urban land ecological security. In this regard, there is an urgent requirement to evaluate the security state of urban land ecosystem for reasonable urban planning and management [5].

### 1.1. Review of Land Ecological Security

The land ecological system is an important component of the terrestrial ecosystem, which is a highly intricate and rapidly changing system. Especially in the urban areas of China, land ecosystem supports natural, societal, economic alterations in a continuous way. Numerous studies suggest land ecology should combine with security, ensuring the proper management of urban land ecosystem [6,7,8]. Most experts define “ecological security” in relation to attempts to safeguard flows of ecological resources, infrastructure and services at the national scale [9]. As an essential component of overall ecological security, land ecological security can also be understood from two points of view: one suggests that the land ecosystem itself is without risk or hazard [10], which refers to a state in which it maintains a healthy and balanced structure and function within certain spatial and temporal ranges; the other implies that the land ecosystem is safe for mankind—that is, the service it provides can satisfy the needs of human beings [11].

Ecological security can be defined as mankind’s security degree un-affected by ecological destruction and environmental pollution in yield, living and health, including basic element of water and food security, air quality and the green environment [12]. Land ecological security evaluation is the fundamental work in this research area, which is both the core and the foundation of the sustainable utilization of land resources [13]. Some researchers consider that LES should focus on the sustainable development promoting the harmonious unity of economy, society and natural environment. Specifically, LES is the complex system composed of land natural-ecological security, land economic-ecological security, and land social-ecological security [14]. Furthermore, LES is also the key reference point for decision-making about land planning, management and protection. Most experts are of the opinion that land ecological security evaluation should evolve from studies of ecological risk and ecological fragility. Excessive resource consumption and exploitation caused great pressure on the ecological system, which rendered high risk to the individuals and human society. Albers and Goldbach (2000) believe that management decisions can bring about discontinuous ecological change, which can lead to economic discontinuities and, in the extreme, a “crash” of both systems [15]. The natural resources management is dramatically essential for human security and society sustainable development according to the study in Cambodia [16]. With increasing human needs, there are large areas of former homelands are destroyed by human activities in South Africa, resulting in a catastrophic reduction in ecosystem function [17]. Recent studies of LES have recommended many theories and methods, however, the standard assessment parameters and methods of ecological security have not been universally accepted [18]. Some studies have been based on fuzzy set pair modeling [19] or catastrophe theory [20]. The pressure-state-response (PSR) [21] and the analytic hierarchy process (AHP) models have also been used to determine LES evaluation indicators—mainly land pressure, land state and environmental response or natural, economic and social ecosystems—and their weighting [22] to quantify regional land ecological security levels. Again, other researchers have explored quantitative spatial changes in land ecosystems and related evolutionary mechanisms to gain an understanding of strategies for regional ecological security [23]. As a complex ecosystem, Bartel (2000) compared deductive method with fuzzy set theory to demonstrate the land ecosystem behavior and the methods applicable [24]. Ecological footprint theory is suitable and widely used for the ecological security or environmental impacts evaluation in worldwide [25,26]. Overall, in recent studies on LES, most of them focused on the regional security situation with various methods and theory, but ignored the unit spatial security in the region.

In practice, “security” to most people evidently means being free from physical threat against, and assaults on, one’s own person; being free from hunger, epidemics, dehydration, disease* etc.*; having shelter from environmental hazards; having a job, an income, financial savings, and a home for life [27]. Security is also a feeling that is based not on objective probabilities and mathematical calculations but on one’s own subjective reactions to both risks and countermeasures [28], and different individuals react differently to threats or risks. On one hand, LES is an objective concept; on the other hand, LES is a personal feeling of city dwellers.

However, most evaluations of land ecological security have ignored people’s feelings and preferences about ecological security, which are the main social factors connected with managing land ecosystems [29,30]. In this paper, we establish the double land ecological security (DLES) model which focuses on the regional and unit evaluation combined with the spiritual feelings of urban residents. It gives a more comprehensive LES evaluation and is meaningful to urban planning and management.

### 1.2. Study Area and Data Preparation

**Figure 1:**
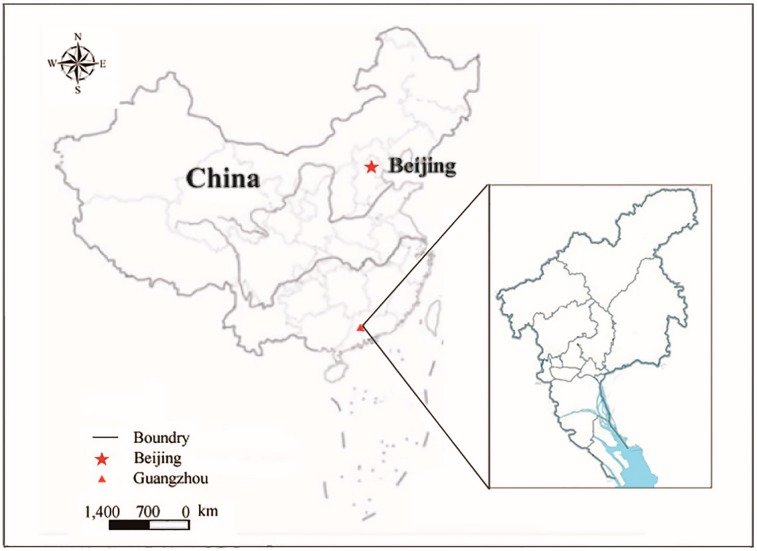
The location of Guangzhou in China.

Guangzhou is one of the most important industrial centers in China, located on the Pearl River (Figure 1), covering an area of 7434 km^2^ and with a population in 2012 of over 12 million. The city of Guangzhou stands at the confluence of the Dongjiang, Xijiang and Beijiang Rivers [31]. The climate is subtropical marine, with an average annual temperature of 21.8 °C, annual rainfall 1694 mm per year, which can affect the productivity and local soil characteristics [32]. Under this climate situation, Guangzhou city generates specific land ecological condition and resources in long term.

Guangzhou is also one of the most developed cities in China, with a first place GDP ranking in Guangdong Province and third place in the whole of China. Its rapid urbanization and industrialization have created serious ecosystem problems, which threatens its land ecological security. Guangzhou was chosen as a case study with the aim of evaluating the land ecological security problems and proposing solutions.

The digital data in China, in this study, were obtained from the Computer Network Information Center, on the Chinese Academy Sciences website [33]. Subsequently, we extracted the information of Guangzhou from the data of China in 2000, 2006 and 2009 respectively.

From 1997 to the present, Guangzhou has carried out two land-use plans: “Overall Land Use Planning in Guangzhou City (1997–2010)” and “Overall Land Use Planning in Guangzhou City (2006–2020)”. The year 2000 is the basic year in the first plan and is also the time that the whole country promoted the concept of Eco-Functional Regionalization. The year 2006 is the start of the second land use plan. As a result, it has access to the various sets of data in the years of 2000 and 2006. The year 2009 is the research time of this study, which represents the present situation of land ecological security at that time. Thus, we choose specific and meaningful data in 2000, 2006 and 2009.

## 2. Methodology

This study proposed a new evaluation method, which integrates physical ecological security concerns and the people’s feelings about the situation, in order to make a crucial and reasonable assessment of land ecological security in regional and unit scales and to provide decision-makers with choices between suitable scenarios for urban land planning.

### 2.1. Evaluation Indices of Land Ecological Security 

Each land use type included nine service function types: food production, raw material, gas regulation, climate regulation, water supply, waste treatment, soil formation and retention, biodiversity protection, and recreation and culture [34]. Land ecological security supply has a relationship with the main functions that the land ecosystem provides: the nurturing function, carrying (or bearing) function, storage function and landscape function [35]. Maslow’s theory of personal motivation suggests that people’s sense of security includes personal safety and health, sufficient resources, adequate property, moral guarantees, job guarantees, family safety, and so on [36]. Clearly, no land ecosystem can directly satisfy all of these individual demands. Recognizing this, we hold that they may be summed up as, firstly, material demand, and secondly, spiritual demand. In addition, we found that the material demand mainly comprises the nurturing, carrying and storage functions of the ecosystem, and the spiritual demand mainly refers to the landscape function (Table 1).

**Table 1:** Evaluation indices of land ecological security (LES).

LES Demand Indices	LES Supply Indices
Material demand (intrinsic demand)	Nurturing function
	Carrying function
	Storage function
Spiritual demand (flexible demand)	Landscape function

#### 2.1.1. LES Demand Indices

***Material demand***. The most basic demands made by humans on the land ecosystem are *space* and *resources*. Urban infrastructure (systems for energy supply, water supply, transport, communication and so forth) are the guaranteed requirement for urban economic and social activities; the land ecosystem is expected to provide space for the infrastructure. Food, mainly produced by the farmland ecosystem, is another basic material demand. Besides these renewable resources, a land ecosystem may also contain energy resources, minerals and other non-renewable resources, all of which are demanded to guarantee the basic viability of people in urban areas. Both the quality and the quantity of these demands are relatively stable. Intrinsic material demands are met by the nurturing, carrying and storage functions of the land.

***Spiritual demand***. Once the material demands have been satisfied, people may seek greater spiritual satisfaction—cultural, educational, leisure, entertainment and other diversions. For example, when the basic food demand has been met, one might wish for a more beautiful natural landscape, fresh air and so on, for relaxation away from daily work. Spiritual demands fluctuate more than material demands, varying between individuals in different economic conditions and at different stages of their lives. Spiritual demands may therefore be regarded as flexible demands of the urban land ecosystem. For land ecosystem in this study, we take landscape function as the main supply for land spiritual security.

#### 2.1.2. LES Supply Indices

***Nurturing function***. Most human activities (e.g., cultivation, habitation, transportation) require space and a suitable substrate (soil) to support the associated infrastructure [37]. Fertile land is beneficial to the growth of crops, providing living materials for both humans and animals, such as food and raw materials from cultivated land and forest.

***Carrying function***. Carrying function mainly refers to the carrier of all buildings, which is one of the mainly functions of the land ecosystem [38]. The provision of places for humans to live and work, and for roads, railways, airports and the like are examples of the carrying function of the land ecosystem.

***Storage function***. Mineral resources (metals, petroleum, coal, natural gas, *etc.*), which are indispensable to modern social and economic development, are mainly extracted from the earth’s crust [35]. This function provides mineral resources for human society.

***Landscape function***. This refers to the way in which ecological resources are regulated and utilized within a landscape [39]. The human sensory system is linked closely with emotions; pleasure has a fundamental influence on our response to the stimuli of our world [40]. The landscape function, then, is valued more for the feelings of comfort and the aesthetics that the land ecosystem provides than for its ecological values.

### 2.2. Land Ecological Security Evaluation Model

#### 2.2.1. Evaluation Framework 

To clarify our concept of land ecological security: in this paper, urban land ecological security is perceived as a problem regarding the extent to which ecological resource supply satisfies human demand for security (in the sense of stability), and establishes an evaluation model of urban land ecological security based on the relationship between supply and the demand that is generated.

**Figure 2:**
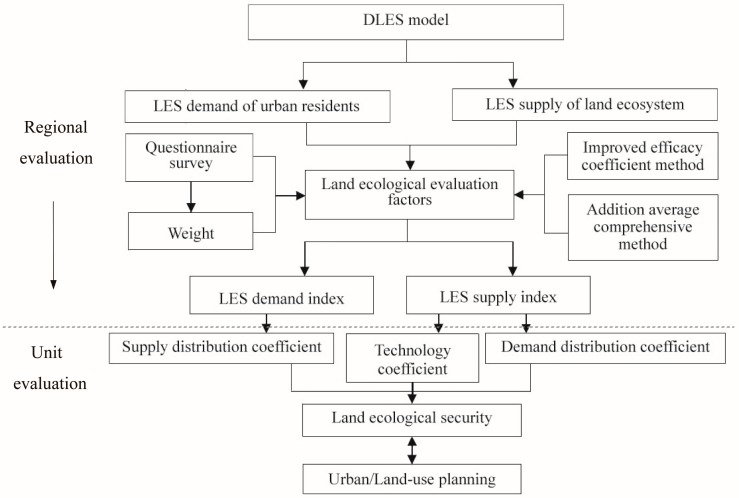
Framework of the DLES model.

We named this the DLES evaluation model, where D refers to the idea of a double-approach evaluation (regional and unit evaluation), and LES is short for “land ecological security”. The DLES model includes both regional and unit evaluation: regional evaluation gives a synthesized and comprehensive assessment of an overall region, reflecting the degree of ecological security from the two aspects of demand and supply, while unit evaluation provides information concerning detailed land ecological pressure and capacity (Figure 2). The unit evaluation introduces supply-distribution and demand-distribution coefficients to reflect, firstly, the different supply capacities of different land ecosystems, and secondly the various individual security demands. In addition, a technology contribution coefficient is proposed to evaluate the LES in unit scale, which represents the influence of scientific and technological developments on improving the quality of land ecosystems.

Land ecological security evaluation focuses mainly on the large scale. In fact, however, the land ecosystem can be considered as an aggregation of uniform land units; on this smaller scale, different types of land satisfy different aspects of security supply and demand. In recognition of this limitation, we have devised an evaluation approach that looks more closely at different scales of urban land ecosystems than others have done.

#### 2.2.2. Evaluation Model of Land Ecological Security on the Regional Scale

To measure regional land ecological security, evaluation indices for measuring the material and spiritual security of the land ecosystem were allocated according to the condition of the study area (Figure 2). Then a questionnaire survey was carried out to ascertain the standard and weight of each index. The questionnaire was designed according to the framework of LES evaluation (Figure 2). It contained five items to ascertain basic information about the respondents, and a further 20 multiple-choice questions focused on the respondents’ demands and reactions about land ecological security. This simple questionnaire was designed to take about 10 min to answer, thus encouraging the respondent to complete it. A total of 450 printed questionnaires were given out, 301 of which were completed. Afterwards, we combined the statistical questionnaire results with other measurement elements to calculate land ecological security supply and demand indices. The details of the calculation are listed as follows.

##### (1) Data Standardization

The data standardization method had to meet the requirement that security demand and supply data were comparable regardless of the time interval over which the comparison was made. The score of security demand indices and security supply indices for different years could be standardized using the improved efficacy coefficient method:

(1) $$a_{ij}=(X_{ij}-X_{jmin})/(X_{jmax}-X_{jmin})\times 0.5+0.5$$

where *a_ij_* is the efficacy coefficient of unit *i* and element *j*; *X_ij_* is the actual value of unit *i* and element *j*; *X_j_*_max_, *X_j_*_min_ are the upper and lower limits of element *j*; *i* = 1, 2, ... ; and *j* = 1, 2,....

##### (2) Comprehensive Calculation of the Regional Security Supply and Demand

The regional security supply and demand indices were calculated using the following equations:

(2) $$SS=\mu \sum_{sx=1}^{sy}\delta_{sx}M_{sx}+\gamma \sum_{sm=1}^{sn}\beta_{sm}V_{sm}$$

(3) $$SD=\mu \sum_{dx=1}^{dy}\delta_{dx}M_{dx}+\gamma \sum_{dm=1}^{dn}\beta_{dm}V_{dm}$$

(4) ULES= SS−SD

where *SS* is the index of regional land ecological security supply; *M_sx_* is the element value of the material security of the land ecosystem; *V_sm_* is the element value of the spiritual security of the land ecosystem; *δ_sx_*, *β_sm_* are the weights of the elements representing material security and spiritual security, respectively; *μ*, *γ* are the weighting of material and spiritual security, which is obtained from the questionnaire survey; *SD* is the index of the regional land ecological security demand; *M_dx_* is the material demand of the land ecosystem; *V_dm_* is the spiritual demand of the land ecosystem; *δ_dx_*, *β_dm_* are the weights of the elements representing material security demand and spiritual security demand, respectively; *ULES* is the regional urban land ecological security.

#### 2.2.3. Evaluation Model of Land Ecological Security in Spatial Units

In this study, the urban ecosystem was divided into a large number of same size units; the supply and demand indices for each unit were then calculated by taking account of the supply/demand distribution coefficient and the technology contribution coefficient (Figure 2). A classification standard was then defined for measuring the land ecological security change spatially.

##### (1) Supply and Demand Distribution

Land ecosystems are complex, consisting of both horizontal and vertical structures, which operate and evolve according to the material cycle and the energy flow between them and other systems [41]. Land ecosystems may be capable of providing living space and resources for use by humans, depending on the function of the particular ecosystem; as examples, the main function of an agricultural ecosystem is to provide food (the nurturing function), and the main function of building land is to provide space for people to live and work (the carrying function). The importance of these functions varies from people, and depends largely on their living conditions: when their living standard is relatively low, food supply is the most imperative function, and the importance to them of an urban greenbelt is relatively low. Thus, in this study, a *supply distribution coefficient* was introduced to take the spatial variability of the security supply into account. The supply distribution coefficient can be expressed as “*R*” in this study, which has a similar meaning to “output factor” in the ecological footprint evaluation [42].

A *demand distribution coefficient* was also established with the aim of highlighting the spatial heterogeneity of human demands. Specifically, when people’s concerns are for their basic life needs, the security demands on the land ecosystem might be purely physical-food, shelter and so on. Once material demands are assured, security demands at the spiritual level may arise, the most important being for the landscape function of the land. The demand distribution coefficient can be expressed as “*D*”, which has a similar meaning to “equivalence factor” in the ecological footprint evaluation [42].

Both supply and demand distribution coefficients’ value depend on the land use category of unit *i*. Referencing the ecological footprint theory, humanity's demand of ecological security is spread across the six land use categories: cropland, grazing land, fishing grounds, forest, built-up area, and land for carbon absorption.

##### (2) Technology Contribution

Technological development contributes greatly to improvements in the production of farmland [43]: examples include crop rotation to reduce the pressure on the land and improve the way existing resources are used; adding fertilizer to supplement natural soil nutrients and produce more food to satisfy the dramatic increases in demand. In recognition of this, a *technology contribution coefficient* was introduced in the present study, given by:

(5) $$T_{agri}=\exp(\frac{{\mathrm{GDP}}_{agri}}{\mathrm{GDP}})$$

where *GDP_agri_* is the agricultural output attributable to the new technology.

##### (3) Calculation of Unit Supply and Demand

The unit supply index is calculated from:

(6) $$\;ULSS_{i}=k\times R_{i}\times T_{agri\;}\times SS$$

where *ULSS_i_* is the supply index of land ecological security of unit *i*; *R_i_* is the supply distribution coefficient of unit *i*, which is calculated on the basis of the main biological production potential and the output factor in the ecological footprint model [44]; *SS* is the regional security supply index obtained from Equation (2); *k* is a constant for numerical standardization, which standardize *ULSS*_i_ data in range of 0–1; and *T*_agri_ is the technology contribution coefficient.

To compare this with security supply, the unit security demand index is calculated as:

(7) $$\;ULSD_{i}=k_{1}\times W\times D_{i}\times SD$$

where *ULSD_i_* is the demand index of land ecological security of unit *i*; *W* is a fluctuation factor, which is a measure the fluctuation of security demand in different economic conditions, defined as the current per-capita GDP divided by the per-capita GDP of the target year; *D_i_* is the demand distribution coefficient of different land use categories, which is determined by the equivalence factor of each land-use type [45]; *k*_1_ is a constant for numerical standardization, which standardize *ULSD_i_* data in range of 0–1; and *SD* is the regional security demand index obtained from Equation (3). The unit ecological security index *ULES_i_* is then the difference between the unit security supply index from Equation (6) and the unit security demand index from Equation (7):

(8) $$\;ULES_{i}=ULSS_{i}-ULSD_{i}$$

##### (4) Classification Standard of Land Ecological Security

Urban land ecological security depends on the capacity of the land ecosystem and the level of demand generated by the urban population. This means that, within a given range of time and space, the land ecosystem can safely meet this demand if the secure ecological supply exceeds the demand, but if the supply is less than the demand then the ecosystem is unsustainable.

After data standardization in this study, ULES ranges from ‒0.5 to 0.5; we divide the value of ULES into four equal levels, which is widely used by researchers in ecological security or risk assessment areas [29,46]. Descriptions of the classification standards are given in Table 2. The maximum value of ULES = 0.5, which indicates that the urban land ecosystem’s structure is complete, function is stable, soil has high fertility, the system can provide good ecological services, human disturbance is slight, and the system has high development potential. When ULES = 0, the security supply is balanced with the security demand, and the urban social economic system will develop according to the existing situation. The minimum value of ULES is –0.5, which refers that urban land supply capacity is almost lost, human demand pressure is great, and the recovery of land-use function is immediately required.

A detailed classification standard was thus developed for that region. The four levels are termed *security*, *marginal security*, *marginal insecurity*, *insecurity*.

**Table 2:** Classification standards of land ecological security.

ULES Level	ULES	Features
Level 1: Security	0.25≤ ULES < 0.5	The security supply is greater than the security demand, the urban land ecosystem’s structure is complete, function is stable, soil has high fertility, the system can provide good ecological services, and ecological problems are not significant.
Level 2: Marginal security	0 < ULES < 0.25	The security supply is greater than the security demand, the land environment is disturbed, and ecological problems are not significant.
Level 3: Marginal insecurity	−0.25≤ ULES <0	The security supply is lower than the security demand, the urban land ecosystem structure is not complete, the function is not stable, soil has low fertility, and ecological problems appear.
Level 4: Insecurity	−0.5 < ULES ≤ −0.25	The security supply is lower than the security demand, and land ecological environment urgently needs to be improved.

## 3. Results and Discussion

### 3.1. Evaluation of Guangzhou on the Regional Scale

In line with to the above urban land ecological security evaluation factor classification, the land ecological security elements for Guangzhou city were classified and, combined with importance scores, the respective evaluation factor weights were calculated.

Because of the constraints on various external resources and the conditions that they impose, the psychological security demand is unstable. In this study, therefore, the security demand fluctuation factor, *W*, was defined as: current per capita GDP/per capita GDP of target year (Table 3).

**Table 3:** Security demand fluctuation factor in Guangzhou.

Year	2000	2006	2009
*W*	0.58	0.78	1.00

Note: Data source: All GDP data obtained from Guangzhou Statistical Yearbooks [47,48,49].

In the questionnaire survey, we asked a question about security feelings of residents. The question for the weight of Demand index in Table 3 is: which one is more important in your personal life security? Options are “material security”, “spiritual security” or “other conditions”. The statistical results showed that 54% of respondents believed that, if the necessary life materials were guaranteed, they considered themselves relatively safe; 44% considered a comfortable life to be their personal security standard; and 2% thought that they also needed other conditions in order to meet their personal security needs. Based on the survey results, the weighting of the material security needs was set at 0.55, and 0.45 for the spiritual security needs, simplified for calculation and analysis.

**Figure 3:**
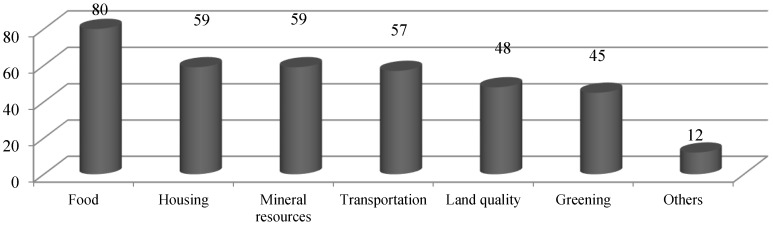
Factor scores of urban land ecological security evaluation.

The question for the weight of Supply index in Table 4 is: to ensure your security, how many scores would you like to give to the following various factors? (Full score is 100) 1. Food; 2. Mineral resources; 3. Housing; 4. Greening; 5. Land quality; 6. Transportation; 7. Others. According to the interviewees’ answers, we found that food, housing, transport and other basic elements, which directly affect residents’ lives, had slightly higher scores in the survey. Land quality and greening, aspects of spiritual satisfaction, scored slightly less (Figure 3).

**Table 4:** Evaluation factors and weights of land ecological security in Guangzhou.

Demand Index (Weight)	Supply Index (Weight)	Element		Weight	Supply							Demand						
					Original data			Standardized Data				Original Data			Standardized Data			
					2000	2006	2009		2000	2006	2009	2000	2006	2009		2000	2006	2009
Material security (0.55)	Carrying (0.32)	Road area per capita (m^2^/person)		0.180	5.67	13.49	10.63		0.56	1.00	0.84	4.60	6.19	7.93		0.50	0.59	0.69
		Residential area per capita (m^2^/person)		0.140	13.13	18.87	21.01		0.61	0.89	1.00	10.97	14.75	18.91		0.50	0.69	0.90
	Nurturing (0.45)	Farmland area per capita (m^2^/person)	Grain (kg/year/person)	0.090	106.71	77.98	64.61		0.95	0.64	0.50	86.88	87.06	111.39		0.74	0.74	1.00
			Vegetables and their products (kg/year/person)	0.090	112.92	104.56	56.49		1.00	0.93	0.50	75.97	97.40	103.01		0.67	0.86	0.91
			Oil and fat (kg/year/person)	0.090	6.87	6.7	4.59		0.84	0.82	0.50	6.18	5.79	7.92		0.74	0.68	1.00
			Meat (kg/year/person)	0.090	47.41	46.37	20.30		0.90	0.88	0.50	27.30	35.00	54.60		0.60	0.72	1.00
			Eggs (kg/year/person)	0.090	8.62	6.24	4.31		1.00	0.72	0.50	5.80	7.43	7.44		0.67	0.86	0.86
	Storage (0.23)	Mining land per capita (m^2^/person)	Coal (tonnes/year 10,000 Yuan GDP)	0.23	0.78	0.65	0.35		0.89	0.77	0.50	0.47	0.60	0.90		0.61	0.73	1.00
Spiritual security (0.45)	Landscape (1)	Rate of urban green coverage (%)		0.245	31.6	36.38	38.21		0.72	0.86	0.91	23.96	32.22	41.31		0.50	0.74	1.00
		Green space area in parks (m^2^/person)		0.245	7.87	11.32	13.76		0.50	0.78	0.97	8.20	11.02	14.13		0.53	0.75	1.00
		Proportion of woodland area (%)		0.255	0.39	0.37	0.34		1.00	0.94	0.84	0.23	0.30	0.39		0.50	0.73	1.00
		Proportion of wetland area (%)		0.255	0.12	0.14	0.18		0.63	0.75	1.00	0.10	0.14	0.18		0.53	0.75	1.00

Notes: 1. Supply data source: *Guangzhou Statistic Yearbook 2000, 2006, and 2009* [47,48,49]. 2. Demand data obtained from questionnaire survey carried out by the authors; security demand for proportion of forestry and wetland determined from the optimal values in 2000, 2006 and 2009.

The data of security demand and supply and its standardized data for the years 2000, 2006 and 2009 are also given in Table 4. The supply index weightings of each category were also calculated based the questionnaire. In the survey, we have listed each category of indices for the interviewees to assign the values of the indices to make them add up to 10, which is relatively convenient for them to answer the questions. Then the average results divided by 10 are the final weights are presented in Table 4.

The regional supply and demand index of land ecological security was calculated from Equations (2) and (3) (Table 5). The material, spiritual supply and demand were obtained from Table 5. The results show that the spiritual security supply of the land ecosystem in Guangzhou has increased in recent years, but the material security supply declined to 0.63 in 2009 causing the security supply index to decrease to around 0.77, which was same to the index in 2000. Corresponding to security supply, material and spiritual security demand also increased speedily year by year. With the rapid pace of urbanization, the material and spiritual demands of the citizens of Guangzhou increased significantly if they were to maintain their personal security standards.

**Table 5:** Regional supply and demand index of land ecological security in Guangzhou.

Year Indices	2000	2006	2009
Ms	0.81	0.84	0.63
ss	0.71	0.83	0.93
SS	0.77	0.84	0.77
Md	0.61	0.72	0.91
sd	0.52	0.74	1.00
SD	0.57	0.73	0.95
ULES	0.20	0.11	−0.18
ULES level	Marginal security	Marginal security	Marginal insecurity

Notes: Ms: material supply; ss: spiritual supply; SS: security supply; Md: material demand; sd: spiritual demand; SD: security demand; ULES: urban land ecological security.

A comparison between security supply and demand in Table 5 shows that LES demand gradually approached and finally exceeded supply in the 10 years 2000–2009: demand outstripped supply by 2009, indicating that the land ecological security of Guangzhou had by then reached a precarious phase where the urban population needed to constrain their activities. In regional scale, the LES index decreased relatively fast from 2000 to 2009, and the security levels dropped from marginal security to marginal insecurity.

In this study, Guangzhou city was considered as a closed system; that is, security supply and demand were related only to the local land ecosystem and consumption by the residents of the city itself, without reference to external influences. Many studies have illustrated that the loss of valuable rural land is very severe with the rapid pace of urbanization. Insufficient farmland is the most prominent problem of the land ecosystem of Guangzhou, with evidence of extensive degradation in farmland. However, because in 2009 there continued to be regular supplies from the rural area, the shortage of farmland had not severely influenced the land ecological security of Guangzhou. This implies that the ecological supply index calculated in this study may be lower than the true value, and the land ecological security of Guangzhou may be higher than this evaluation suggests. Nevertheless, natural resources in the rural area are limited, and the increasing security demand of the citizens threatens the local land ecological security.

### 3.2. Evaluation of Guangzhou in Spatial Units

This study divided Guangzhou into a grid comprising about 28,504 units measuring 500 m × 500 m for computing the unit supply index. The land ecological security unit supply index is calculated by the regional supply index multiplied by the supply distribution coefficient and technology contribution coefficient.

#### 3.2.1. Unit Supply Index of Land Ecological Security

##### (1) Supply Distribution Coefficient

Recognizing that both the spatial scale and the quality classification of the different land-use types affect the calculated supply capacity, the supply distribution coefficient in this study was calculated using a method of segmented variable weighting (Table 6).

**Table 6:** Supply distribution coefficients for different land-use covers.

Year	Farmland	Garden	Woodland	Grassland	Built-up	Wetland
2000	2.24	1.20	1.20	3.29	2.24	1.00
2006	1.48	1.69	1.12	1.62	2.69	1.12
2009	1.22	1.86	1.11	1.63	2.94	1.53

##### (2) Spatial Differentiation of Unit Supply Index

**Figure 4:**
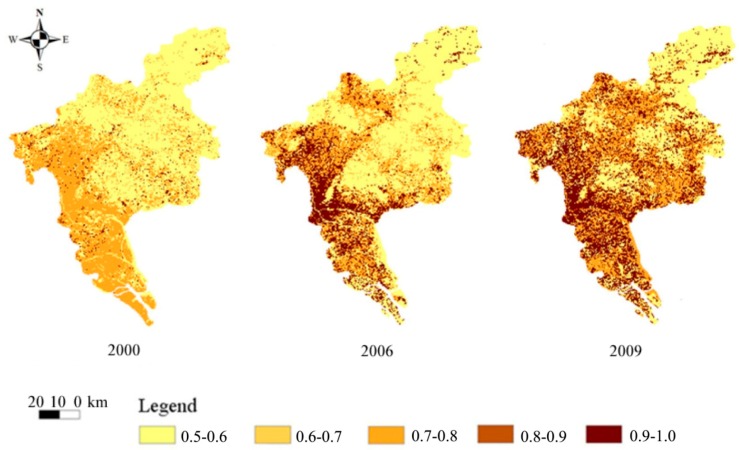
Spatial differentiation of unit supply index.

The evaluations of spatial differentiation of unit supply index for the years 2000, 2006 and 2009 are shown in Figure 4, where it can be seen that the supply index of the land ecological security gradually increased from 2000 to 2009. The high supply index values are concentrated in the central, southern and north-western areas of Guangzhou. Well-built infrastructures in the central and north-western areas provide basic space and resources. The southern district contains large areas of rich farmland that provide ample food for the population; these are important function zones. In the 10 years leading up to 2009, the security supply ability of the central area of Guangzhou gradually improved as the city developed.

The spatial supply indices for 2000, 2006 and 2009 are summarized in Table 7, which shows the number of units in different index values. The unit supply index mainly lay in the 0.6–0.9 range; for any one of the three years the supply index of most individual units was 0.6–0.7. The number of units with a given range decreased as the unit supply index increased. From the time sequence, the number of units in the range 0.6–0.7 gradually declined from 2000 to 2009. The number of units in the 0.7–0.8 range firstly decreased, and then increased slightly. The number of units in the 0.8–0.9 range increased year by year.

**Table 7:** Unit supply index of land ecological security in Guangzhou city.

Year	0.5–0.6	0.6–0.7	0.7–0.8	0.8–0.9	0.9–1.0
2000	–	16,928	10,759	808	–
2006	–	16,071	7441	5018	–
2009	–	10,663	8841	9019	–

Note: “–” means that there is no land unit in the relevant range.

#### 3.2.2. Unit Demand Index of Land Ecological Security

The unit demand index was calculated as regional demand index multiplied by demand distribution coefficient.

##### (1) Demand Distribution Coefficient

The demand distribution coefficient measures the spatial differences in security demand that occur because of the variation in the type of demand and the demand requirements from one land ecosystem to another. The dynamic segmented variable weight was used to manifest the differences in the demand distribution coefficient at different times (Table 8).

**Table 8:** Demand distribution coefficients for different land-use covers.

Year	Farmland	Garden	Woodland	Grassland	Built-Up	Wetland
2000	2.80	1.10	1.10	0.50	2.80	0.20
2006	1.85	1.55	1.03	0.25	3.36	0.22
2009	1.53	1.71	1.01	0.25	3.67	0.31

##### (2) Spatial Differentiation of Unit Demand Index

The evaluation results for 2000, 2006 and 2009 showed that the demand index of the land ecological security of Guangzhou fluctuated greatly. For a single year, the demand index of most units in the grid was in the 0.6–0.7 range. From 2000 to 2009 the index gradually increased: in 2000, the index in many of the units was concentrated in the 0.5–0.7 range; in 2006, in some areas values increased to 0.7–0.9; and in the year 2009, some values even reached the 0.9–1.0 range (Table 9).

**Table 9:** Unit demand index of land ecological security in Guangzhou city.

Year	0.5–0.6	0.6–0.7	0.7–0.8	0.8–0.9	0.9–1.0
2000	17736	10759	–	–	–
2006	4034	17497	1981	5018	–
2009	3264	10663	5577	–	9019

Note: “–” means that there is no land unit in the relevant range.

**Figure 5:**
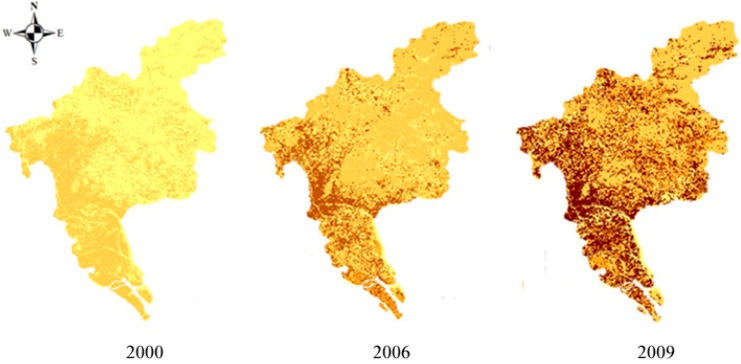
Spatial differentiation of unit demand index.

The changes in the land ecological security demand index for Guangzhou are illustrated in Figure 5. Temporally, the land ecological security demand of Guangzhou was generally low in the year 2000; spatially, the security demand in the northeastern area was relatively high, and lower in the southwest. This might have been due to the fact that the farmland is mainly distributed in the south-western areas. In 2006, the demand was clearly stronger than in 2000, and strengthened further in 2009, mainly in the central and southern areas. In summary, between 2000 and 2009, the land ecological security demand increased continuously, with enhanced demand intensity for each land-use type, and a greater annual demand area.

#### 3.2.3. Technology Contribution Coefficient

The technology contribution coefficient defined in Equation (5) was proposed mainly as a measure of the effect of utilizing scientific and technological developments to improve land productivity and ecosystem services. The technology contribution coefficients from 2000 to 2009 are listed in Table 10. The numbers show a slightly increasing trend.

**Table 10:** Technology contribution coefficient in Guangzhou.

Year	2000	2006	2009
*T*_agri_	1.0037	1.0047	1.0049

Note: Data source: All GDP data were obtained from the Guangzhou’s Statistical Yearbooks [47,48,49].

#### 3.2.4. Unit Comprehensive Index of Land Ecological Security

To compare land ecological security supply and demand, four levels of security were established: *security*, *marginal security*, *marginal insecurity* and *insecurity*.

The ecological security comprehensive index showed that the number of units in levels of security, marginal-security and marginal-insecurity (Table 11). The unit number of marginal insecurity increased while the number of unit gradually decreased in the levels of marginal security and security.

**Table 11:** Unit comprehensive index of land ecological security in Guangzhou.

Year	Insecurity	Marginal-Insecurity	Marginal-Security	Security
2000	–	–	10,759	17,736
2006	–	6999	8439	13,092
2009	–	9019	8841	10,663

Note: “–” means that there is no unit in the certain level.

**Figure 6:**
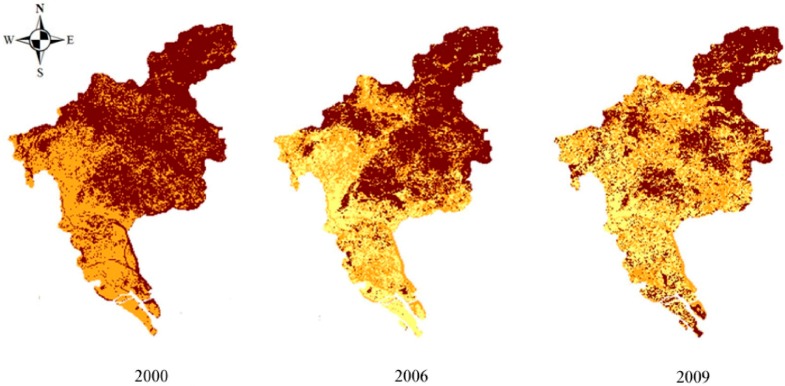
Spatial differentiation of unit comprehensive index.

Figure 6 shows the spatial differentiation of land ecological security index for Guangzhou in 2000, 2006 and 2009. It is seen that the pre-2006 index ranged between marginal-security and security and, spatially, that the southern and eastern parts of Guangzhou were in a state of marginal-security; after 2006, however, some of the southern and eastern areas were in a marginal-insecurity state. The spatial differentiation between security supply and security demand indicated that the supply capability was relatively strong in central and eastern Guangzhou, but the demand pressure was also high in these regions. Because the demand pressure was approaching the secure supply values, the overall trend of ecological security gradually decreased into insecurity.

Spatially, the ecological security zone was mainly concentrated in the north and in some regions in the east of the province. These regions, where the land ecosystem function was high, were important ecological sources, and represented a higher state of land ecological security. Land ecological security supply capability in the southern and central areas of Guangzhou was strong, but was also under large demand pressure, so the region gradually changed from ecological security to insecurity.

### 3.3. Results Comparison with International Studies

In China, rapid pace of urbanization causes severe ecosystem degradation in wide-range cities; therefore, many studies illustrated the land ecological fragile and insecurity state in recent years. Due to the different development styles and the amount of land resources, in this research, most areas in Guangzhou city were in the marginal security and marginal insecurity in 2009, while the study of Xiamen showed that the overall LES was in medium-high level, and increased in 2006 compared with the results of 2003 [50]. Guangzhou suffered a shortage of security supply, including material supply and spiritual supply, while Lianyungang, as a bay area in China, was in a quite different situation, which had a widespread urban–rural interface with rapid land-use changes and wetland degradation since 2005 when the eastern coastal development policy was implemented [51]. Instead of evaluation the ecological security in regional scale [29], this study established a double LES evaluation model (DLES) for the regional and unit scales ecological evaluation based on the supply and demand equivalence. Most land ecological security studies take land ecosystem as a material supporter [52,53] and ignore the spiritual supply for human, thus we combine the spiritual feelings into the DLES model for comprehensive and holistic evaluation of the land ecological security, aiming at providing an effective planning and management guidance for urban sustainable development.

## 4. Conclusions

Our research has produced an in-depth analysis of the theory of land ecological security by integrating the traditional evaluation of land ecological security with psychological security, to establish an urban land ecological security evaluation model based on a supply-and-demand balance in regional and unit scales. The LES evaluation is closely related to the land use types, which could give reasonable propositions and instructions for the land use planning and prediction under the land ecological security target. The DLES model was applied to assess the land ecological security of Guangzhou, China.

In this study, land ecological security is the capability of the land ecosystem to provide continuous and stable ecological supply to the human population in reaction to demand, the relationship being a function of security supply and security demand, including material and spiritual aspects.

Spiritual security accounted for about 45% of the overall land ecological security. This means that the spiritual security is an important part which should not be ignored in many studies, especially when the material supply satisfies people demand.

Different areas with various land ecological functions have different supply capabilities. Therefore, in unit scale LES evaluation, the results illustrated that there were obvious spatial variation of land ecological security. The unit LES evaluation can provide more detailed information about the LES situation, which can make urban planning and management be more targeted than the regional evaluation.

Analysis of the results of evaluation the LES in Guangzhou implies that the security demand and the security supply both show an increasing trend as a whole. The land ecological security of the city is tending to decrease, which will influence the local people and the sustainable development of the city in the long run. What is more, most of the southern and central parts are in marginal security. Because of this, reasonable and appropriate urban ecological planning and some other measures need to be put in force.

Above all, we propose that the DLES model be used for land ecological security evaluation (including material and spiritual security) and be actively integrated into the urban planning and management, which could provide more authentic LES evaluation results for decision making.
